# Chemicals of concern in personal care products used by women of color in three communities of California

**DOI:** 10.1038/s41370-022-00485-y

**Published:** 2022-11-02

**Authors:** Paula I. Johnson, Kristin Favela, Jennifer Jarin, Amy M. Le, Phyllis Y. Clark, Lisa Fu, April D. Gillis, Norma Morga, Caroline Nguyen, Kim G. Harley

**Affiliations:** 1 California Safe Cosmetics Program, California Department of Public Health, Richmond, CA USA; 2 Southwest Research Institute, San Antonio, TX USA; 3 Center for Environmental Research and Children’s Health (CERCH), School of Public Health, University of California Berkeley, Berkeley, CA USA; 4 Healthy Heritage Movement, Riverside, CA USA; 5 California Healthy Nail Salon Collaborative, Oakland, CA USA; 6 The Center for the Health Assessment of Mothers and Children of Salinas (CHAMACOS) Study at Clinica de Salud del Valle de Salinas, Salinas, CA USA

**Keywords:** Chemicals in Products, Cancer, Analytical Method, Endocrine Disruptors, Environmental Justice, Vulnerable Populations

## Abstract

**Background:**

Personal care products (PCPs) may contain chemicals associated with adverse health effects. Prior studies found differences in product use by race/ethnicity and suggest some women are disproportionately exposed to chemicals of concern (CoCs).

**Objective:**

We quantified chemicals linked to cancer, reproductive or developmental harm, or endocrine disruption in PCPs used by women of color.

**Methods:**

We documented PCPs in stores frequented by Black, Latina, and Vietnamese women in their communities in California and CoCs on ingredient labels of 546 unique hair, skin, makeup, nail, deodorant/perfume, and intimate care products. Community partners chose 31 products for a combined targeted and suspect screen (National Institute of Standards and Technology mass spectral library search) two-dimensional gas chromatography time-of-flight mass spectrometry (GCxGC-TOFMS) analysis to detect chemicals not on ingredient labels.

**Results:**

We found that 65% of labels included CoCs, and 74% of labels had undisclosed ingredients listed as “fragrance.” The most prevalent chemicals were parabens, cyclosiloxanes, and formaldehyde releasers. GCxGC-TOFMS found additional CoCs, including fragrances, solvents, preservatives, ultraviolet filters, and contaminants.

**Significance:**

These findings contribute to awareness of potentially hazardous chemicals in PCPs, can help estimate disparities in chemical exposure, and complement research on health inequities due to chemical exposures from various contributors.

**Impact statement:**

This study is one of the first detailed assessments of chemicals of concern found in various types of PCPs used by several racial/ethnic groups. We found that over half of the 546 products selected by community partners as marketed to and/or used by them contained ingredients linked to cancer, reproductive or developmental harm, or endocrine disruption. Laboratory analysis identified additional chemicals in a subset of products, including unlabeled fragrance chemicals and contaminants. Elucidating exposures to chemicals in PCPs is important for risk assessment and health inequity research.

## Introduction

Personal care products are used widely and are a significant source of exposure to chemicals, some of which are associated with an array of health effects, including cancer and disruption of the endocrine and reproductive systems [1, 2]. People may be exposed to chemicals in personal care products directly through dermal absorption, inhalation, and ingestion, and possibly indirectly, through contamination of the indoor environment. Frequency of product use is associated with body burdens of chemicals commonly found in personal care products. For instance, phthalates and parabens are common additives in personal care products, and the use of a greater number of products was associated with higher urine concentrations of these chemicals or their metabolites [3]. Women reporting a higher use of perfume had nearly three times the urine concentrations of the metabolite of diethyl phthalate [4]. More frequent use of makeup is associated with higher urinary parabens and metabolites of phthalates, as was use of sunscreen and benzophenone-3 (BP-3) [5]. Positive associations were also found between use of liquid soap and triclosan, and lotion and parabens [6]. Switching to personal care products labeled to be free of phthalates, parabens, triclosan, and BP-3 resulted in lower urine concentrations in Latina adolescents, indicating that exposure can be controlled to some extent by informed product choices [7].

There is evidence of racial/ethnic differences in exposures to chemicals that are found in personal care products. In a national U.S. survey, urinary levels of some phthalates and parabens were higher in African American women, compared to White women [8]. Other analyses show that both African Americans and Mexican Americans have higher urinary phthalates and parabens than Whites, and that Asians have the highest levels of triclosan [9–11].

Black women have a higher incidence of hormone-mediated health outcomes such as preterm birth [12]. There are also racial/ethnic differences in the prevalence of early puberty, a risk factor for breast cancer [13]. There are well-established racial disparities of breast cancer. For example, in the United States, Black women have a higher rate of premenopausal breast cancer, more aggressive types of cancer, and are 40% more likely to die of breast cancer than White women [14]. Eurocentric beauty standards and discrimination based on perceived odor, hair texture, and skin tone have been identified as drivers of racial/ethnic differences in personal care product choice and use, and this may contribute to differences in chemical exposures and health disparities [15]. U.S. Black women report higher use of scented intimate care products, which is associated with higher urinary metabolites of diethyl phthalate [16] and higher blood levels of 1,4-dichlorobenzene and ethylbenzene, which can be fragrance additives [17]. Childhood use of hair oil and hair perm, which is higher among African Americans, is associated with earlier age of menarche [18]. Hair dye and relaxer/straightener use is associated with incidence of breast cancer, particularly in Black women [19, 20]. There is less research on product use patterns among Latinas and Asian women. Occupational exposures to chemicals in nail salon products have been studied in Vietnamese women [21, 22], but assessment of personal product use is very limited. One study of Chinese women found that a higher level of acculturation in the U.S. was associated with higher use of several types of personal care products [23].

Studies of chemicals in personal care products are limited, particularly studies that examine specific product types used by different races/ethnicities. A survey of U.S. women found the highest use of hair products among African Americans and African Caribbeans and, according to ingredient labels, their products were more likely to contain endocrine disrupting chemicals (EDCs) [24]. Six of the products identified from the survey were found to be hormonally active, with some having estrogenic properties, which is of concern for hormone-mediated diseases such as breast cancer [25]. Helm et al. [26] measured EDCs and asthma-associated chemicals in 18 hair products identified in the same survey and found that many of these chemicals were not disclosed on the product label. A separate analysis of 25 personal care products documented the presence of carcinogens, EDCs, respiratory toxicants, and developmental toxicants, and found that nearly 80% of those chemicals were fragrance ingredients exempt from labeling requirements [27].

Previously, we surveyed specific communities in California and found differences in the types of products and the frequency of use by race/ethnicity [28]. We found that Black/African American women used fragrances and certain hair products or styles most frequently, Latinas used makeup most frequently, and Vietnamese women were most likely to use facial cleansing products than other women. In the present study, we examined ingredient labels of products marketed to or used by Latinas and Black/African American and Vietnamese women in these communities. Although some research has been conducted on personal care product chemicals, there is much that is still unknown about the extent to which known or suspected carcinogens, reproductive or developmental toxicants, or endocrine disruptors are found in products, particularly products used by women of color, and the extent to which they are exposed to these chemicals. In the present study, we employed targeted analysis of specific chemicals along with suspect screening to detect chemicals not identified on labels. Suspect screening is increasingly used for the evaluation of the chemical composition, or signatures, of many different sample types [29–31]. Other investigation of consumer products, including personal care products, used comprehensive two-dimensional gas chromatography–time of flight mass spectrometry (GCxGC-TOFMS) and resulted in the detection of over 4,000 unique chemical signatures across 100 products and brought attention to the large amount of uncharacterized and unknown chemicals in consumer products [32]. We utilized the same analytical method in the present study, where we conducted an analysis of product ingredient labels and laboratory testing, in one of the first detailed assessments of potentially hazardous chemicals found in various types of personal care products differentially used by several racial/ethnic groups.

## Methods

### Chemicals of concern

We defined chemicals of concern (CoCs) as substances appearing on any of several lists of substances determined by scientific entities to be associated with, suspected, or known to cause cancer, reproductive or developmental harm, or endocrine disruption. The lists consulted were: (1) The California Safe Cosmetics Program Reportable Ingredients List [33] (includes substances identified as being known or suspected to cause cancer, birth defects, or other developmental or reproductive toxicity by (a) the California Environmental Protection Agency’s Office of Environmental Health Hazard Assessment Proposition 65 List, (b) the U.S. Environmental Protection Agency, (c) the National Toxicology Program’s (NTP) Report on Carcinogens, (d) the NTP Office of Health Assessment and Translation, or (e) the International Agency for Research on Cancer (IARC)); (2) Silent Spring Institute Mammary Gland Carcinogen Database; [34] (3) Mammary gland developmental toxicants from Rudel et al. [35]; (4) European Union Candidate List of Substances of Very High Concern in accordance with Article 59 of Regulation (EC) 1907/2006 on the basis of Article 57(f) for endocrine disrupting properties [36]; and (5) the list of endocrine disrupting substances compiled by The Endocrine Disruption Exchange (TEDX) [37]. We included all the potential endocrine disruptors identified by TEDX because this list is precautionary, i.e., inclusive of substances with limited evidence, but we also considered evaluation by the European Chemicals Agency (ECHA) [38] as a means to differentiate the level of concern for those substances. In addition, we included formaldehyde-releasing preservatives because formaldehyde is a CoC (known human carcinogen, see Table 1).

**Table 1:** Chemicals of concern (CoCs) identified on labels or by laboratory analysis in personal care products.

Chemical name (alternate names)	CAS RN	Health concern			Evidence (CoC List)	Typical use^19^
		Cancer	Developmental/reproductive toxicity	Endocrine disruption		
CoCs found on labels						
Ammonium chloride	12125-02-9			x	1	Buffer
Avobenzone	70356-09-1			x	1, 3	UV absorber
Benzophenone-1	131-56-6			x	1	UV absorber
Benzophenone-3 (Oxybenzone)	131-57-7			x	1, 3	UV absorber
Beta-carotene	7235-40-7			x	1	Colorant
Butylated hydroxyanisole (BHA)	25013-16-5	x		x	1, 7, 8	Antioxidant
Butylparaben	94-26-8			x	1, 5	Preservative
Caffeine	58-08-2			x	1	Conditioner
Carbon black (D&C black no. 2)^15^	1333-86-4	x			7, 8	Colorant
Cocamide diethanolamine (Cocamide DEA)	68603-42-9	x			7, 8, 10	Emulsifier/Surfactant
Cyclopentasiloxane	541-02-6			x	1, 2	Conditioner/Emollient
Cyclotetrasiloxane	556-67-2			x	1, 2	Conditioner/Emollient
Diazolidinyl urea	78491-02-8	x			14	Preservative
Dimethyloldimethyl hydantoin (DMDM hydantoin)	6440-58-0	x			14	Preservative
Hexamethylindanopyran (Galaxolide)	1222-05-5			x	1, 2, 4	Fragrance
Hydroquinone	123-31-9			x	1, 6	Antioxidant/Bleaching
Imidazolidinyl urea	39236-46-9	x			14	Preservative
Isobutylparaben	4247-02-3			x	1, 12	Antimicrobial
2-Methylresorcinol	608-25-3			x	1	Hair dye
Oleic Acid	112-80-1			x	1	Conditioner/Emollient
Polyoxymethylene urea	68611-64-3	x			14	Bulking agent
Propylparaben	94-13-3			x	1, 3	Preservative
Resorcinol	108-46-3			x	1, 3	Antioxidant/Hair dye
Retinol/retinyl esters^16^	68-26-8		x		8	Conditioner
Selenium sulfide	7446-34-6	x			8	Anti-dandruff
Sodium hydroxymethylglycinate	70161-44-3	x			14	Preservative
Talc^17^	14807-96-6	x			7, 8	Absorbent
Titanium dioxide^15^	13463-67-7	x			7, 8	Colorant
Tocopherol, Tocopheryl acetate	1406-18-4			x	1	Antioxidant
Triphenyl phosphate	115-86-6			x	1, 3	Plasticizer
CoCs found by laboratory analysis						
Benzaldehyde	100-52-7			x	1	Denaturant
Benzoic acid^18^	65-85-0			x	1	Preservative
Benzophenone	119-61-9	x			7, 8	UV absorber
Benzyl chloride^20^	100-44-7	x			7, 8, 10, 12	Contaminant
Butylated hydroxytoluene (BHT)^18^	128-37-0			x	1, 3	Antioxidant
Butylphenyl methylpropional (Lilial)^18^	80-54-6		x	x	1, 3, 9, 12	Fragrance
Celestolide	13171-00-1			x	1	Fragrance
Cyclohexanone	108-94-1			x	1	Fragrance
Dibutyl phthalate (DBP)	84-74-2		x	x	1, 2, 5, 8, 9, 11, 12	Fragrance/Plasticizer
Diethylhexyl adipate	103-23-1			x	1	Plasticizer
Diethyl phthalate (DEP)	84-66-2			x	1, 3	Fragrance/Plasticizer
Diethylhexyl phthalate (DEHP)	117-81-7	x	x	x	1, 5, 7, 8, 9, 10, 12	Solvent/Plasticizer
Diisobutyl phthalate (DIBP)^20^	84-69-5			x	1, 5, 9	Plasticizer
1,4-Dioxane^20^	123-91-1	x			7, 8, 10, 12, 13	Contaminant
Estragole	140-67-0	x			8	Fragrance
2-Ethyl hexanol	104-76-7			x	1	Fragrance
Ethylbenzene	100-41-4	x			7, 8	Fragrance
Ethylparaben^18^	120-47-8			x	1, 3	Preservative
Homosalate^18^	118-56-9			x	1	UV absorber
Isopropylparaben	4191-73-5			x	1, 12	Preservative
Methyl salicylate	119-36-8			x	1, 3	Denaturant/Fragrance
Methylparaben^18^	99-76-3			x	1, 3	Preservative
Musk ketone	81-14-1				1, 6	Fragrance
β-Myrcene	123-35-3	x			7, 8	Fragrance
Octinoxate (Octyl methoxycinnamate)^18^	5466-77-3			x	1	UV absorber
Pulegone	89-82-7	x			7, 8	Fragrance
Safrole^20^	94-59-7	x			7, 8, 13	Fragrance
Tonalid	21145-77-7			x	1, 3	Fragrance
Versalide^20^	88-29-9			x	1, 12	Fragrance

^1^TEDX list of potential endocrine disruptors.

^2^European Union candidate list of Substances of Very High Concern for persistent, bioaccumulative and toxic (PBT), or very persistent and very bioaccumulative properties in accordance with Article 59 of Regulation (EC) 1907/2006 on the basis of Article 57(d), Article 57(e), or Article 57(f).

^3^Of concern and under assessment for endocrine disruption by European Chemicals Agency.

^4^Of concern and under assessment as PBT by European Chemicals Agency.

^5^European Union candidate list of Substances of Very High Concern in accordance with Article 59 of Regulation (EC) 1907/2006 on the basis of Article 57(f) for endocrine disrupting properties.

^6^Suspected carcinogen or mutagen by European Chemicals Agency.

^7^International Agency for Research on Cancer (IARC).

^8^California Proposition 65 List.

^9^European Union Candidate List of Substances of Very High Concern in accordance with Article 59 of Regulation (EC) 1907/2006 on the basis of Article 57(c) for reproductive toxicity.

^10^United States Environmental Protection Agency Integrated Risk Information System (IRIS).

^11^United States National Toxicology Program Office of Health Assessment and Translation.

^12^European Union Cosmetic Products Regulation, Annex II - Prohibited Substances.

^13^United States National Toxicology Program Report on Carcinogens.

^14^Formaldehyde releasing compounds did not appear on CoC lists, but formaldehyde does appear as a carcinogen (lists 7, 8, 10, 13).

^15^Concern when respirable.

^16^Concern when in daily dosages in excess of 10,000 IU, or 3,000 retinol equivalents.

^17^Concern when used in perineal area (e.g., body powder) or if contaminated with asbestos or asbestiform fibers.

^18^This CoC also appeared on some product labels, but was found in a greater percentage of products analyzed by lab.

^19^Typical use was noted from the European Commission Cosmetic Ingredients (CosIng) database unless otherwise specified (https://ec.europa.eu/growth/tools-databases/cosing).

^20^Typical use was noted from PubChem (https://pubchem.ncbi.nlm.nih.gov).

### Selection of products and ingredient label review

This was a community-based study that included a partnering organization from each of the racial/ethnic communities of interest in California: Healthy Heritage Movement, serving the Black/African American community in San Bernardino and Riverside counties; Clinica de Salud del Valle de Salinas, serving the Latino community in Salinas Valley; and California Healthy Nail Salon Collaborative, serving Vietnamese communities throughout California and focusing on Los Angeles and Orange counties for this study. Product selection occurred through a qualitative process guided by three considerations: community survey findings, store inventories, and knowledge of common use. Community partners used these considerations to assess popular product use and availability, and they ultimately chose the final list of products to review. First, we prioritized product types for selection based on use frequencies from our community survey [28]. Then, we conducted a store inventory of products between November 2019 and early March 2020. Community partner representatives visited 39 different stores across their three respective communities. They aimed to visit a full range of store types, from large, well-known franchises to independent community markets (Table S1). Community representatives photographed products that were marketed to their race or ethnicity as indicated by images, certain color schemes (e.g., darker brown colored bottle marketed to Black women), or phrases on the products, or if the product was labeled in a language other than English. We documented all brands, product names, and product types in the photographs from each store in a master spreadsheet.

We identified the most common products found across all stores in each community and prioritized these products for ingredient label review. Community partners included additional products based on their knowledge of popular products used in their communities and did not necessarily select products from each product category. We conducted ingredient label reviews of 546 products between April and December of 2020. We found the product labels online, documented ingredients in a database, corrected any typos, and used a Microsoft Excel-based macro to automate the task of comparing each product’s ingredients against a compiled list of chemicals and their commonly used synonyms to identify chemicals of concern in each product. We also recorded instances of “fragrance” or “parfum” listed in ingredient labels because those terms indicate additional unidentified ingredients added to products. We summarized ingredient frequencies in descriptive statistics (Microsoft Excel) for all products and by race/ethnicity group and product category.

### Laboratory analysis

Due to cost restraints, we selected only 31 products for laboratory analysis to screen for additional CoCs not identified on product labels. We prioritized products for analysis by reviewing the labels and selecting products containing “fragrance” or ingredients known to be commonly contaminated with chemicals such as 1,4-dioxane. We chose ten products from each community, including two products that did not have an ingredient label available, and one product that was marketed as “pure,” “safe,” and “natural” by a known alternative “natural” brand. Community partners made the final decision on which products to analyze based on their knowledge of what types of products were of most concern to their constituents. Due to the beginning of the COVID-19 pandemic, we purchased products online rather than revisit the stores and shipped directly to the laboratory.

Two-dimensional gas chromatography time-of-flight mass spectrometric (GCxGC-TOFMS) analysis was performed using an Agilent 7890 gas chromatograph coupled to a LECO PEGASUS 4D-TOF (LECO, St. Joseph, MI). High-resolution GCxGC-TOFMS analysis was performed using an Agilent 7890A gas chromatograph coupled to a LECO HRT (LECO, St. Joseph, MI). Sample preparation and method details, including a list of target analytes (Table S2), can be found in the Supplementary Information.

Calibration curves for chemicals either anticipated to be present or of concern for their presence in consumer products were assayed with the first pass data collection. No corrections for surrogate recovery were applied. Chemicals detected below the lowest calibration standard are reported as “trace.” The reported concentration of benzoic acid is considered estimated given poor chromatographic performance.

For suspect screening, data was processed using LECO’s Chromatof software to integrate peaks and to identify them against the National Institute of Standards and Technology (NIST 2017) library. Data were passed through the Floodlight program, described elsewhere, for machine learning removal of artifacts and poor-quality peaks [39]. All remaining, high-quality signals were manually reviewed and classified as either unknowns or tentatively identified compounds. From the list of tentatively identified compounds, we identified 20 compounds of interest for confirmation using either a single-point standard or high-resolution mass spectrometry, according to reference standard availability (Table S3). Concentrations of chemicals with single-point calibration (retention time-only standards) were calculated by comparison of peak area response to the internal standard naphthalene-D8. Therefore, although the identity is confirmed, the concentrations are estimated.

### Reporting summary

Further information on research design is available in the Nature Research Reporting Summary linked to this article.

## Results

### Ingredient labels

Table 1 describes the CoCs identified in this study, either on product labels or by laboratory analysis and indicates why each chemical meets our CoC definition and the use for which the chemical is typically added to a product formulation. We reviewed the ingredient labels of 546 unique personal care products. We found 30 unique CoCs that appeared only on product labels. Through lab analysis of 31 products, we found 29 additional unique CoCs, 7 of which also appeared on product labels in some instances.

Table 2 lists the CoCs found on all 546 product labels by order of frequency. Products containing CoCs had from 1 to 10 CoCs, with an average of 1.2 CoCs per product (data not shown). We found a total of 37 unique CoCs on product labels. Tocopherol, or tocopheryl acetate, synonymous to vitamin E, had the highest frequency and appeared in mostly skin care products. Because this ingredient has only limited evidence for endocrine disruption and is an essential nutrient, we excluded it from further analysis of selected CoCs. Titanium dioxide had the second highest frequency, but because its classification as a possible human carcinogen is based on inhalation of respirable particles, we only included it in further analysis of makeup powders. Talc appeared on labels of 18 products, but only one of these was a body powder. Perineal use of talc-based body powder is considered possibly carcinogenic by IARC [40]. Of the remaining instances of talc, only 7 were in inhalable powdered (makeup) products; however, it is unknown whether the talc in these products is contaminated, which is the basis for IARC’s distinct listing of talc containing asbestos or asbestiform fibers as carcinogenic. We only included talc in inhalable products in further analysis of selected CoCs.

**Table 2:** Chemicals of concern listed on ingredient labels (*n* = 546 products).

Chemical name	Products listing these ingredients	
	*N*	%
Tocopherol/Tocopheryl acetate	198	36.3
Titanium dioxide	81	14.8
Methylparaben	74	13.6
Cyclopentasiloxane	56	10.3
Butylated hydroxytoluene (BHT)	50	9.2
Propylparaben	49	9.0
DMDM hydantoin	44	8.1
Butylphenyl methylpropional/Lilial	43	7.9
Benzoic acid	34	6.2
Retinol/Retinyl palmitate	24	4.4
Talc	18	3.3
Ethylparaben	16	2.9
Diazolidinyl urea	15	2.7
Imidazolidinyl urea	13	2.4
Avobenzone	11	2.0
Caffeine	10	1.8
Oxybenzone/Benzophenone-3	7	1.3
Benzophenone-1	6	1.1
Butylated hydroxyanisole (BHA)	5	0.9
Carbon black	5	0.9
Homosalate	5	0.9
Octinoxate/Octyl methoxycinnamate	5	0.9
Beta-carotene	4	0.7
Cyclotetrasiloxane	4	0.7
Ammonium chloride	3	0.5
Butylparaben	3	0.5
Hexamethylindanopyran (Galaxolide)	3	0.5
Hydroquinone	3	0.5
Oleic acid	3	0.5
Triphenyl phosphate	3	0.5
Cocamide DEA	2	0.4
Isobutylparaben	2	0.4
Resorcinol	2	0.4
Sodium hydroxymethylglycinate	2	0.4
2-Methylresorcinol	1	0.2
Polyoxymethylene urea	1	0.2
Selenium sulfide	1	0.2

We examined products from 6 different categories (hair, skin, makeup, nail, deodorants/perfumes, and intimate care products), although there was not an equal spread of products from each category across community groups (Table 3). CoCs varied by product type. Hair products had the greatest number of unique CoCs or groups of CoCs, followed by skin products, although these product types were also the highest represented products overall, 203 and 238 products, respectively (Table 3). Parabens were the most frequent group of chemicals identified on product labels, and different parabens usually co-occurred in the same product (49 of 81 products). We found butylated hydroxyanisole (BHA), a less common preservative listed as a carcinogen on California’s Proposition 65 (Prop 65) list, on labels of five hair products used by Black women. Two other Prop 65 carcinogens, cocamide diethanolamine (cocamide DEA) and selenium sulfide, were on labels of shampoos used by Latinas. We also found cocamide DEA on the label of one body wash (skin care) used by Latinas. The fragrance ingredient lilial, which is a reproductive toxicant prohibited by the European Union, was common in hair and skin products. Hydroquinone, a suspected endocrine disruptor and carcinogen used as a skin bleaching agent, was on the label of one skin lightening cream used by Black women and two skin lightening creams used by Latinas. Titanium dioxide was a common ingredient in makeup, listed on more than 73% of labels of inhalable makeup products such as face powders (Table 3). We found parabens and BHT on labels of makeup products used by Latinas, but none on labels of makeup products used by Vietnamese women. Of the 23 intimate care product labels we examined, including washes, sprays, powders, moisturizers, douches, and wipes, over 60% had undisclosed fragrance ingredients. We found undisclosed fragrance ingredients on 85% of all deodorant or perfume product labels, and the specific fragrance ingredients, lilial and galaxolide, were each listed on 10% of deodorant/perfume products. We found undisclosed fragrance ingredients and triphenyl phosphate, a plasticizer and potential endocrine disruptor, on 3 of 9 nail product labels we examined.

**Table 3:** Selected^a^ Chemicals of Concern (CoCs), by Product Category and Community.

	Black	Latina	Vietnamese	Total
Total products, *n*	154	180	212	546
Products containing any CoCs	71%	63%	62%	65%
Selected CoCs	*n* (%)	*n* (%)	*n* (%)	*n* (%)
Fragrance/parfum^b^	143 (92.9)	140 (77.8)	120 (56.6)	403 (73.8)
Parabens^c^	32 (20.8)	32 (17.8)	17 (8.0)	81 (14.8)
Formaldehyde releasers^d^	27 (17.5)	37 (20.6)	8 (3.8)	72 (13.2)
Cyclosiloxanes^e^	17 (11.0)	18 (10.0)	21 (9.9)	56 (10.3)
BHT	16 (10.4)	17 (9.4)	17 (8.0)	50 (9.2)
Lilial	20 (13.0)	12 (6.7)	11 (5.2)	43 (7.9)
Total hair products, *n*	112	46	45	203
Selected CoCs	*n* (%)	*n* (%)	*n* (%)	*n* (%)
Fragrance/parfum^b^	104 (92.9)	41 (89.1)	44 (97.8)	189 (93.1)
Parabens^c^	19 (17.0)	4 (6.5)	3 (6.7)	26 (12.8)
Formaldehyde releasers^d^	19 (17.0)	10 (21.7)	6 (13.3)	35 (17.2)
Cyclosiloxanes^e^	14 (12.5)	5 (10.9)	3 (6.7)	22 (10.8)
Lilial	20 (17.9)	4 (8.7)	6 (13.3)	30 (14.8)
BHT	10 (8.9)	1 (2.2)	4 (8.9)	15 (7.4)
BHA	5 (4.5)	0 (0)	0 (0)	5 (2.5)
Cocamide DEA	0 (0)	1 (2.2)	0 (0)	1 (0.5)
Selenium sulfide	0 (0)	1 (2.2)	0 (0)	1 (0.5)
Oxybenzone	3 (2.7)	2 (4.3)	0 (0)	5 (2.5)
Avobenzone	0 (0)	0 (0)	2 (4.4)	2 (1.0)
Resorcinol	0 (0)	0 (0)	2 (4.4)	2 (1.0)
Hexamethylindanopyran (Galaxolide)	0 (0)	0 (0)	1 (2.2)	1 (0.5)
Retinol/retinyl palmitate	6 (5.4)	2 (4.3)	0 (0)	8 (3.9)
Total skin products, *n*	36	93	109	238
Selected CoCs	*n* (%)	*n* (%)	*n* (%)	*n* (%)
Fragrance/parfum^b^	29 (80.6)	76 (81.7)	55 (50.5)	160 (67.2)
Parabens^c^	13 (36.1)	18 (19.4)	12 (11.0)	43 (18.1)
Formaldehyde releasers^d^	8 (22.2)	22 (23.7)	1 (0.9)	31 (13.0)
BHT	6 (16.7)	10 (10.8)	11 (10.1)	27 (11.3)
Cyclosiloxanes^e^	3 (8.3)	4 (4.3)	5 (4.6)	12 (5.0)
Lilial	0 (0)	7 (7.5)	3 (2.8)	10 (4.2)
Retinol/retinyl palmitate	3 (8.3)	5 (5.4)	5 (4.6)	13 (5.5)
Cocamide DEA	0 (0)	1 (1.1)	0 (0)	1 (0.4)
Oxybenzone	1 (2.8)	0 (0)	1 (0.9)	2 (0.8)
Avobenzone	1 (2.8)	3 (3.2)	4 (3.7)	8 (3.4)
Hydroquinone	1 (2.8)	2 (2.2)	0 (0)	3 (1.3)
Talc	0 (0)	1 (1.1)	0 (0)	1 (0.4)
Total makeup products, *n*	0	24	29	53
Selected CoCs	*n* (%)	*n* (%)	*n* (%)	*n* (%)
Fragrance/parfum^b^	N/A	3 (12.5)	2 (6.9)	5 (9.4)
Titanium dioxide	N/A	15 (62.5)	24 (82.8)	39 (73.6)
Parabens^c^	N/A	10 (41.7)	0 (0)	10 (18.9)
Cyclosiloxanes^e^	N/A	7 (29.2)	8 (27.6)	15 (28.3)
BHT	N/A	3 (12.5)	0 (0)	3 (5.7)
Formaldehyde releasers^d^	N/A	2 (8.3)	1 (3.4)	3 (5.7)
Talc	0 (0)	4 (16.7)	2 (6.9)	6 (11.3)
Total intimate care products, *n*	6	11	6	23
Selected CoCs	*n* (%)	*n* (%)	*n* (%)	*n* (%)
Fragrance/parfum^b^	3 (50.0)	8 (72.7)	3 (50.0)	14 (60.9)
Parabens^c^	0 (0)	0 (0)	2 (33.3)	2 (8.7)
Formaldehyde releasers^d^	0 (0)	2 (18.2)	0 (0)	2 (8.7)
Lilial	0 (0)	1 (9.1)	0 (0)	1 (4.3)
Total deodorant/perfume products, *n*	0	6	14	20
Selected CoCs	*n* (%)	*n* (%)	*n* (%)	*n* (%)
Fragrance/parfum^b^	N/A	6 (100)	11 (78.6)	17 (85.0)
Cyclosiloxanes^e^	N/A	2 (33.3)	4 (28.5)	6 (30.0)
BHT	N/A	3 (50.0)	2 (14.3)	5 (25.0)
Lilial	N/A	0 (0)	2 (14.3)	2 (10.0)
Formaldehyde releasers^d^	N/A	1 (16.7)	0 (0)	1 (5.0)
Hexamethylindanopyran (Galaxolide)	N/A	0 (0)	2 (14.3)	2 (10.0)
Total nail products, *n*	0	0	9	9
Selected CoCs	*n* (%)	*n* (%)	*n* (%)	*n* (%)
Fragrance/parfum^b^	N/A	N/A	3 (33.3)	3 (33.3)
Cyclosiloxanes^e^	N/A	N/A	1 (11.1)	1 (11.1)
Triphenyl phosphate	N/A	N/A	3 (33.3)	3 (33.3)

*N/A* is not applicable—community partners did not always select products from every product category.

^a^“Selected” chemicals of concern include chemicals with a higher level of evidence for concern; does not include chemicals that appeared only on the TEDX list of potential endocrine disruptors and does not include products with chemicals that have certain exposure caveats that would likely not be met for the product category (titanium dioxide, carbon black: see Table 1). We included inhalable products containing talc due to the potential of contamination by asbestos or asbestiform fibers.

^b^“Fragrance” or “parfum” on a product label represents any number of unidentified ingredients, some of which may be carcinogens, reproductive or developmental toxicants, or endocrine disruptors.

^c^Products containing one or more of the parabens listed in Table 1.

^d^Products containing any of the formaldehyde releasers listed in Table 1.

^e^Products containing one or more of the cyclosiloxanes listed in Table 1 (cyclopenta- or cyclotetrasiloxane).

Among all products, 65% had CoCs listed on ingredient labels, and 74% had undisclosed fragrance ingredients, i.e., the label listed only “fragrance” or “parfum” rather than identify the chemicals composing the fragrance mixtures (Table 3). Higher percentages were found among products used by Black women; 71% contained CoCs and 93% had undisclosed fragrance ingredients., although product types varied across racial/ethnic groups. We found “fragrance” or “parfum” listed on the majority of labels in every product category except for makeup and nail products, where it was listed in only a portion of labels. Among hair products, fragrance appeared on over 93% (189 of 203 total hair products) of labels across all race/ethnic community groups.

Of the 39 stores we visited to find products for review, 21 stores sold some products with labels printed only in a language other than English, despite the English language labeling requirement of U.S. federal law. Products labeled in Spanish had English information online or were translated by community partners. Fifty-two product labels in other languages, e.g., Japanese or Korean, were not found in English online and we were unable to review their ingredients.

### Laboratory analysis

**Fig. 1:**
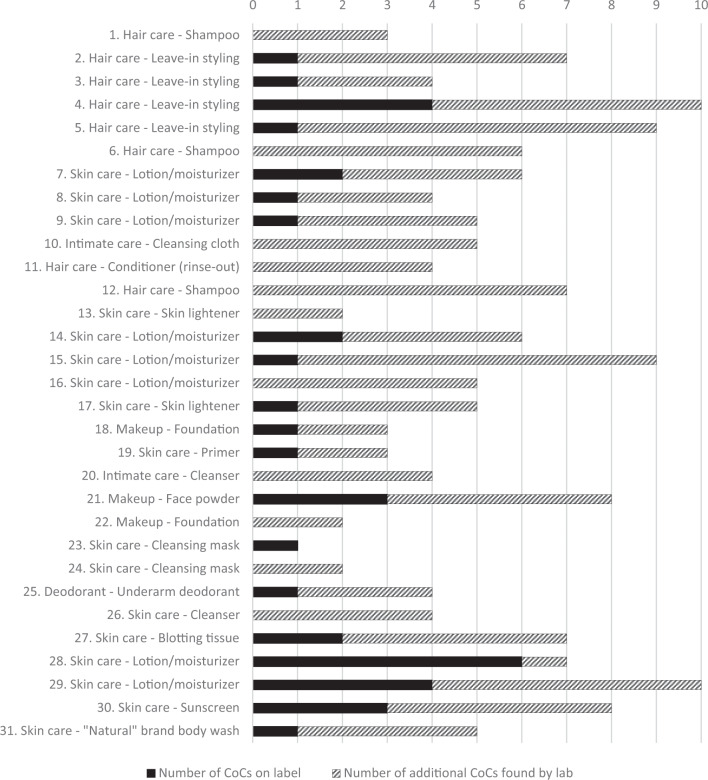
Number of chemicals of concern in 31 products analyzed. Each number in the vertical axis represents a unique product, for a total of 31 products. Products 1-10 were from the Black/African American community, Products 11-20 were from the Latina community, and Products 21-30 were from the Vietnamese community. Product 31 was not from any of the partner communities but was marketed as “natural” and “safe” and for use by anyone. Product #16 and #24 did not have ingredient labels.

We analyzed 31 products by GCxGC-TOFMS, including shampoos, hair styling creams, face cleansers and creams, body lotions, deodorant, makeup, and intimate care products. We found a total of 29 CoCs in the 31 products: 22 additional CoCs not identified on product labels, plus 7 CoCs that appeared on some labels, 1 of which (ethylparaben) always appeared on the label. All 31 products contained from 1 to 10 CoCs, but only 23% of these chemicals (38 of 165 detections) were listed on ingredient labels (Fig. 1). Eleven products did not show any CoCs on labels, but laboratory analysis revealed 2 to 7 CoCs in every one of these products (Fig. 1).

The majority of CoCs found by laboratory analysis were fragrance ingredients (Table 1), typically used to provide scent [41]. Other CoCs found are typically used as preservatives, solvents, plasticizers, or ultraviolet light (UV) absorbing agents, and may be added to stabilize the fragrance mixture (Table 1). Two carcinogens, benzyl chloride and 1,4-dioxane, found in 5 and 3 products respectively, are typically unintended contaminants when found in personal care products [42]. We found 1,4-dioxane, a known contaminant of intentional ingredients added to products as foaming agents, in a shampoo, an intimate wash, and a moisturizer (Table 4). We measured benzyl chloride up to 39 μg/g product in a leave-on skin lotion.

**Table 4:** Concentration of chemicals of concern in personal care products selected by three communities.

	Black/African American community										Latina community										Vietnamese community										N/A	Detections	% Detections
	Intimate care—cleansing cloths	Hair care—leave—in styling	Hair care—shampoo	Hair care—leave—in styling	Hair care—leave—in styling	Hair care—leave—in styling	Hair care—shampoo	Skin care—lotion/moisturizer	Skin care—lotion/moisturizer	Skin care—lotion/moisturizer	Skin care—lotion/moisturizer	Hair care—conditioner	Intimate care—wash/cleanser	Hair care—shampoo	Skin care—lotion/moisturizer	Skin care—lotion/moisturizer	Skin care—lotion/moisturizer	Skin care—primer	Skin care—skin lightener	Skin care—skin lightener	Skin care—blotting tissues	Make-up—foundation	Make-up—face powder	Skin care—cleanser	Skin care—cleansing mask	Skin care—cleansing mask	Deodorant	Skin care—lotion/moisturizer	Skin care—lotion/moisturizer	Skin care—sunscreen	Skin care—“natural” soap/bodywash		
Target chemicals (μg/g sample)																																	
Dibutyl phthalate (DBP)	–	–	–	–	–	–	–	–	–	–	trace	–	trace	–	–	–	–	–	–	–	Trace	–	–	–	–	–	–	–	–	–	–	3	9.7
Diethyl phthalate (DEP)	0.1	–	–	44	–	–	–	–	–	–	–	–	–	8.1	–	1,431	–	–	–	–	–	–	–	–	–	–	–	–	–	–	–	4	12.9
Diethylhexyl phthalate (DEHP)	–	1019	–	–	–	–	–	–	–	–	–	–	–	–	–	–	trace	–	–	–	Trace	–	–	–	–	–	–	–	–	–	–	3	9.7
Diisobutyl phthalate (DIBP)	–	–	–	–	–	–	–	–	–	–	–	–	–	–	–	–	–	–	–	–	Trace	–	–	–	–	–	–	–	–	–	–	1	3.2
1,4—Dioxane	–	–	–	–	–	–	–	–	–	–	–	–	31	12	–	1.7	–	–	–	–	–	–	–	–	–	–	–	–	–	–	–	3	9.7
Ethylparaben	–	–	–	–	–	–	–	2170	–	–	–	–	–	–	–	–	–	–	–	–	–	–	–	–	–	–	–	1228	–	–	–	2	6.5
Isopropylparaben	–	–	–	–	–	–	–		–	1051	–	–	–	–	–	778	–	–	–	223	–	–	–	–	–	–	–	–	–	–	–	3	9.7
Methylparaben	–	–	–	–	–	–	–	2537	–	1332	–	–	–	–	–	1328	–	–	–	867	–	–	2210	–	1024	–	–	1161	–	1062	–	8	25.8
Octinoxate (Octyl methoxycinnamate)	–	–	–	–	–	207	–	–	–	–	–	–	6.5	–	–	–	–	–	–	195	–	–	–	–	–	–	–	–	–	–	–	3	9.7
Non—targeted chemicals (estimated μg/g sample)																																	
Benzaldehyde	0.001	0.2	0.2	–	–	1.5	0.06	–	24	0.1	–	6.1	–	71	–	–	–	–	0.5	–	–	0.3	–	–	0.1	–	1.7	–	0.04	–	25	15	48.4
Benzoic acid	128	0.4	–	64	60	96	6.8	13	118	1.6	–	–	291	–	–	1.3	–	–	–	–	–	–	–	1.0	–	–	–	–	–	–	–	12	38.7
Benzophenone	–	1.0	–	–	–	–	0.3	–	–	–	–	–	–	–	–	–	–	–	–	–	0.04	–	–	–	–	–	–	–	–	6.5	–	4	12.9
Benzyl chloride	–	0.4	–	–	–	–	0.6	–	39	–	–	–	–	2.2	–	–	–	–	–	–	–	–	–	–	–	–	–	–	–	–	0.5	5	16.1
Butylated hydroxytoluene (BHT)	–	3.3	50	347	–	0.1	–	4.2	116	1.3	–	–	–	–	120	1.8	–	–	–	12	–	–	–	–	–	–	157	–	0.2	183	0.5	14	45.2
Butylphenyl methylpropional (Lilial)	–	–	–	39	–	–	–	–	–	5.1	31	14	–	26	–	–	–	–	–	–	–	–	1.6	–	–	–	–	–	19	–	–	7	22.6
Celestolide	–	–	–	–	–	–	–	–	–	–	–	–	–	1.0	–	–	–	0.8	0.2	–	–	–	–	–	–	–	–	–	–	–	–	3	9.7
Cyclohexanone	–	–	–	–	–	–	–	–	–	–	–	–	–	–	–	–	–	–	–	–	0.02	–	–	–	–	–	–	–	–	–	–	1	3.2
Diethylhexyl adipate	–	0.4	–	16	–	–	–	–	–	–	4.3	0.2	0.1	–	–	–	0.1	–	1.8	0.5	0.1	–	–	0.3	–	–	0.1	–	–	0.2	–	12	38.7
Estragole	–	–	–	–	–	–	–	–	–	–	–	–	–	–	–	–	–	–	–	–	–	–	–	1.9	–	–	–	–	4.7	–	–	2	6.5
2—Ethyl hexanol	0.01	–	–	–	–	–	–	1.5	–	–	1	–	–	–	–	–	0.9	0.2	0.2	–	1.3	1.6	–	–	–	–	–	–	–	17	–	9	29.0
Ethylbenzene	–	–	–	–	–	–	–	–	–	–	–	–	–	–	–	–	–	–	–	–	0.1	–	–	–	–	–	–	–	0.2	–	–	2	6.5
Homosalate	0.003	–	–	–	–	–	0.3	–	–	–	–	–	–	–	–	–	–	–	–	–	–	–	–	–	–	–	–	–	–	30,915	–	3	9.7
Methyl salicylate	–	–	–	–	–	–	–	–	–	–	–	–	–	–	–	–	–	–	–	–	–	–	–	–	–	–	–	–	–	16	–	1	3.2
Musk ketone	–	–	–	3.1	–	–	–	–	–	–	–	–	–	–	–	–	–	–	–	–	–	–	–	–	–	–	–	–	–	–	–	1	3.2
β—Myrcene	–	4.9	–	3.5	2.7	1.4	0.2	0.4	–	–	3.1	–	–	2.3	–	0.1	–	–	0.3	0.3	–	–	0.6	7.7	–	–	1.0	–	0.7	2.6	12	17	54.8
Pulegone	–	–	–	–	1.7	–	–	–	–	–	–	–	–	–	–	–	–	–	–	–	–	–	–	–	–	–	–	–	–	–	–	1	3.2
Safrole	–	–	0.3	–	–	0.3	–	–	–	–	–	–	–	–	3,275	–	–	–	–	–	–	–	–	–	–	–	–	–	–	–	–	3	9.7
Tonalid	–	–	–	6.1	–	–	–	–	–	–	–	–	–	–	–	–	–	–	–	–	–	–	–	–	–	–	–	–	–	–	–	1	3.2
Versalide	–	–	–	25	78	113	378	–	–	–	1.8	11	–	1.7	–	10	–	–	–	–	–	–	8.2	–	–	–	–	5.9	7.3	–	–	11	35.5

Each column represents a single product. The total number of products analyzed for each product type are: Intimate care (2); Hair care (8); Skin care (18); Makeup (2); Deodorant (1). “–” indicates chemical not detected.

Of the 29 CoCs found by lab analysis, 9 were target analytes for which we confirmed identity by comparison to NIST standards (Table 4). We detected dibutyl phthalate, an endocrine disruptor and reproductive and developmental toxicant, in trace levels only in three products. We measured diethyl phthalate (DEP), a suspected endocrine disruptor, in four products, at up to 1431 μg/g in a leave-on skin moisturizer used by Latinas. We measured diethylhexyl phthalate (DEHP), a carcinogen and reproductive/developmental toxicant that is prohibited from use in European cosmetics, at 1019 μg/g in a leave-on hair styling serum used by Black women and in trace levels in two other products. We detected diisobutyl phthalate, a suspected endocrine disruptor and reproductive toxicant used as a plasticizer, in only one product (skin blotting tissue used by Vietnamese women) at trace level. We measured parabens in eight various products applied to the skin, ranging from lotions to a makeup powder. Although we detected methylparaben most frequently, more than one paraben often appeared together in the same product, with concentrations of any one paraben ranging from 223 to 2537 μg/g. We detected the UV-absorbing additive, octyl methoxycinnamate, which has limited evidence of endocrine disruption, in a leave-on hair styling product, an intimate wash, and a skin lightening cream.

We detected the remaining 20 CoCs through suspect screening. We detected beta-myrcene, a fragrance ingredient listed as possibly carcinogenic by IARC, most frequently (18 of 31 products). Beta-myrcene is naturally occurring in some plants used to produce fragrances, and the product with the highest concentration of beta-myrcene (over 12 μg/g) was the body wash marketed as “natural.” We detected a total of four CoCs in this same product, including benzyl chloride (Table 4). We found the UV filter, homosalate, on ingredient labels of only sunscreen products (*n* = 5), while the lab analysis identified homosalate in a shampoo, intimate care cloths, and at 30,915 μg/g (3%) in a sunscreen used by Vietnamese women. This concentration may be underestimated by the laboratory method; the sunscreen product listed 10% homosalate. We detected diethylhexyl adipate, a phthalate substitute with limited evidence of endocrine disruption, in a greater number of products (12 of 31) as compared to phthalates. We detected one or more fragrance ingredients in all but four products, despite that three of these products listed the term “fragrance” on the ingredient label. The fourth product did not have an ingredient label available online for comparison.

## Discussion

We analyzed ingredient labels of 546 personal care products marketed to and/or used by Black/African American women, Vietnamese women, and Latinas in our partner communities and found that over half of these products contained ingredients linked to cancer, reproductive or developmental harm, or endocrine disruption.

The most prevalent CoCs were parabens, cyclosiloxanes, and formaldehyde releasers. By laboratory analysis, we found additional CoCs, including fragrances, solvents, preservatives, ultraviolet filters, and contaminants. We found high percentages (74% overall) of labels did not disclose ingredients used as fragrance, across all racial/ethnic groups and nearly all product categories.

There is no comprehensive authoritative list of endocrine disruptors. The TEDX list includes potential endocrine disruptors, and the strength of the evidence and the potential risk posed by these CoCs varies. Of the 59 total CoCs we found either on product labels or by laboratory analysis, 15 of them appeared only on the TEDX list. When omitting one of these chemicals that was also an essential nutrient (tocopherol), we found that 56% of products still had one or more CoCs on the label (compared to 65%). Endocrine disruption is still an overlooked concern, particularly in the regulatory domain, despite hormones being central to numerous body functions and disruption contributing to major diseases [43, 44].

Cumulative exposure to the same or similarly acting chemicals from frequent use of multiple products may have additive effects and result in higher-than-expected exposures. A recent survey in California found that women used a median of 8, and up to 30, personal care products daily [45]. We found a total of 37 CoCs listed on labels of 546 products, and up to 10 CoCs listed on a single product. We found that some of the same CoCs, particularly the more frequently occurring ones (parabens, formaldehyde releasers, and cyclosiloxanes) appeared on labels across all or most product types. More research is needed on formaldehyde-releasing preservatives to better evaluate toxicity and how consumers may be exposed to formaldehyde during product use [46].

We tested a subset (31 products) using GC-MS and found additional chemicals of concern not identified on product labels. The majority of CoCs found by laboratory analysis were ingredients used as fragrance. Others are typically used as preservatives, solvents, plasticizers, or UV-absorbing agents, but all of these are likely components of the fragrance mixture added to the product when not listed on the ingredient label. There were three products with no fragrance CoCs detected despite having the term “fragrance” on the ingredient label. This indicates that there are alternate fragrance ingredients that are not CoCs, or that they are not detectable by GC-MS. Some of the chemicals we found (phthalates, parabens) are being phased out of products [47]. We found diethylhexyl adipate, an alternative to some phthalates, more frequently than we found phthalates. There is some evidence of endocrine disruption for diethylhexyl adipate, but it is not currently on government authoritative lists.

Few studies of personal care products exist for which to compare the concentrations of CoCs we measured. Helm et al. [26] measured methylparaben up to 2100 μg/g in hair lotions used by Black women, which is similar to the highest concentration we measured (2537 μg/g) in skin lotion used by Black women in our communities. We found parabens in seven skin products and one makeup product but did not find parabens in the four leave-on hair styling products we tested. Helm et al. also measured DEP up to 2448 μg/g, which is on the same order of magnitude we measured in skin lotion, but we measured only 44 μg/g DEP in a hair lotion. Helm et al. measured up to 90 μg/g DEHP, while we measured 1019 μg/g DEHP in a leave-on hair lotion. Both studies found levels of octinoxate at the same order of magnitude in leave-on hair lotions.

A 2013 survey of phthalates and parabens in 170 personal care products detected phthalates most frequently in leave-on products, and up to 7980 μg/g of DEP in perfume [48]. The study measured up to 52.3 μg/g DEP among 23 skin lotions, while we measured 1431 μg/g DEP in a skin lotion marketed to and used by Latinas. The same survey also found parabens in 60% of leave-on products, up to 3540 μg/g, which is on the same order as our maximum level (2537 μg/g) of methylparaben in a skin lotion. Another study measured the fragrance ingredients galaxolide and tonalid in perfumes, body lotions, and deodorants, up to 451 μg/g tonalid [49]. We found galaxolide on product labels (*n* = 3), but we did not detect it in the 31 products we tested. We detected tonalid in one product, a leave-on hair styling lotion used by Black women, at 6.1 μg/g.

Dodson et al. [50] tested an array of consumer products in 2012, including 50 types of personal care products, and found several EDCs that were not listed on product labels, including various phthalates, parabens, and fragrance ingredients. They found levels of methylparaben up to 1600 μg/g in sunscreen and in other personal care products that did not list parabens on the label. We found comparable levels of phthalates for similar products, although Dodson et al. found higher levels in makeup (foundation) and perfume (up to 14,000 μg/g) [50]. They also found that both conventional and several products marketed as alternative or natural contained parabens and phthalates. We tested one product, a body wash, that was marketed as “natural” and “pure” and did not detect parabens or phthalates, but detected four other CoCs, including the carcinogenic contaminant benzyl chloride.

Our study was limited in the number of product labels we reviewed, considering the vast number of products available. We surveyed a total of only 39 stores to come up with a list of products used by women in our partner communities, thereby limiting the products representative of those used by certain races/ethnicities. Due to convenience and efficiency of review, we relied on online ingredient labels, which have the potential to change and possibly incorrectly represent the ingredients of a product that is in the store. We did not examine any products of brands that are sold online only, but our prior community survey suggested that the women in these communities primarily shopped in stores represented in our store inventories.

The different communities chose different product types to review, limiting our ability to draw conclusions based on racial/ethnic differences in chemicals within the same product type. However, we examined a large number of products among all three groups. We also did not examine a group of products not specifically marketed to or used by women of color, thereby limiting our conclusions about differences between certain races/ethnicities and White women, or differences between targeted and mainstream products. In addition, products with non-English labels were underrepresented in our review because we were unable to examine labels for which translations were not available. Among the products selected for label review, there was minimal overlap between community groups. For example, the Black and Latina community selected the same brand of skin lotion but different variations; the Black community’s product was a “cocoa” scent and was packaged in a dark brown bottle. Among all products selected independently by each community group, we found a total of 3 hair products and 1 skin care product that were duplicated between groups.

Laboratory screening for unidentified chemicals was limited to chemicals that can be analyzed by GCxGC-TOFMS, and could underestimate the total number of CoCs, e.g., the method we employed would not find heavy metal contaminants, highly volatile chemicals such as formaldehyde, or highly non-volatile chemicals more amenable to liquid chromatography. Other than comparing to product labels, we did not investigate the source of the chemicals found by laboratory analysis. Potential sources could include not only undeclared ingredients, but possibly starting material impurities, processing, and packaging (e.g., plasticizers). Another important limitation to our study and others on consumer product chemical exposure and toxicity is that some chemicals have little or no toxicological information available. Toxicological evaluation does not keep up with the demand and commercialization of new formulations. In addition, there is very limited study of the toxicity of chemicals of concern in combination with other chemicals or of the same chemical from cumulative sources or routes of exposure. Studies that measure the concentrations of CoCs in products can be used to inform dose levels for toxicological studies and exposure assessments. Lastly, our focus was cancer, reproductive/developmental harm, and endocrine disruption, and we did not survey or test for the presence of chemicals associated with other health endpoints such as asthma or allergies. Therefore, we expect that these products would have a greater number of CoCs if we expanded our scope.

Personal care products, or cosmetics, have limited regulation in the United States. Federal law requires intentionally added ingredients to be listed on product labels, but fragrance and flavor ingredients are exempt [51]. Products sold in the U.S. are also required to be labeled in English, although we found several products in our partner communities that were not compliant. Previously, we found that a portion of women in the surveyed racial/ethnic groups had concerns about certain ingredients in their products, with phthalates, parabens, formaldehyde, and fragrance being named most commonly [28]. It can be difficult for consumers to avoid exposure to CoCs if they do not know the hazards and if ingredients are not fully disclosed. The state of California recently passed laws that are more stringent than federal laws. The Cosmetic Fragrance and Flavor Ingredient Right to Know Act of 2020 commenced in 2022 and requires companies selling personal care products in California to report to the state products with fragrance or flavor ingredients that have been identified by authoritative bodies to have known or suspected health effects, including carcinogens, reproductive or developmental toxicants, endocrine disruptors, neurotoxicants, respiratory sensitizers, allergens, and persistent, bioaccumulative, or toxic pollutants [52]. The reported product and ingredient information is displayed for the public on the California Safe Cosmetics Program product database [53]. Another new law, The Toxic Free Cosmetics Act, commences in 2025 and aligns with the European Union by banning nine chemicals and three chemical groups from cosmetics sold in California, including two phthalates, two parabens, formaldehyde, and several perfluoroalkyl substances (PFAS) [54].

## Conclusions

We analyzed the ingredient labels of a wide range of personal care products marketed to and/or used by Black/African American women, Vietnamese women, and Latinas, and found that over half of the products contain ingredients linked to cancer, reproductive or developmental harm, or endocrine disruption. The majority of products contain fragrance ingredients that are not disclosed on product labels, and laboratory analysis identified these chemicals and others that are of concern to consumers’ health.

Our findings can be combined with product use frequency data and inform risk assessments incorporating use of multiple products and exposure routes, with the goal of reducing exposures, particularly for communities most at risk. Further research should investigate how products targeted to certain groups compare with mainstream or untargeted products and should examine disparities in the context of affordability of safer products. Moreover, fully automated tools can be used to efficiently screen ingredients of larger numbers of products against an expanded scope of chemicals of concern. Lack of ingredient disclosure inhibits informed consumer purchasing decisions. More complete ingredient disclosure and greater awareness of the hazards of chemicals in personal care could steer the market toward safer alternatives.

## Data Availability

All data are presented within this paper, its tables and figure, and its Supplementary File.

## Supplemental information

Supplemental information
Reporting Summary
