# Supplementary material for: Chemicals of concern in personal care products used by women of color in three communities of California
Source: J Expo Sci Environ Epidemiol. 2022 Nov 2;32(6):864–76. doi: 10.1038/s41370-022-00485-y (PMC9628299; doi:10.1038/s41370-022-00485-y)
Supplement: Supplementary file 1 — Supplemental information [file 41370_2022_485_MOESM1_ESM.docx]

**Supplemental to Chemicals of Concern in Personal Care Products Used by Women of Color**

Paula I. Johnson, Kristin Favela, Jennifer Jarin, Amy M. Le, Phyllis Y. Clark, Lisa Fu, April D. Gillis, Norma Morga, Caroline Nguyen, and Kim G. Harley

This Supplemental file contains: a list of stores visited by community partners for an “inventory” of products (Table S1); a description of the sample preparation for the GCxGC-TOFMS analysis and the instrument parameters; tables containing the reference standards calibration curves, i.e., target analytes (Table S2); and the chemicals confirmed using retention time standard or measured by high resolution mass spectrometry (Table S3).

Table S1. Store Inventory Locations

| **Community Partner** | **Store Name** | **Store Type** |
| --- | --- | --- |
| California Healthy Nail Salon Collaborative | Ulta Beauty | Beauty Supply Store |
|  | Skyline Beauty Supply | Beauty Supply Store |
|  | Palace Beauty Galleria | Beauty Supply Store |
|  | Sally Beauty Supply | Beauty Supply Store |
|  | Beauty Zone | Beauty Supply Store |
|  | Walmart | Big Box Store |
|  | Target | Big Box Store |
|  | Macy's | Department Store |
|  | Skylark Nail & Beauty Supply | Local/Community Store |
|  | Make Asobi | Local/Community Store |
|  | Maneki Neko | Local/Community Store |
|  | Victoria's Secret/PINK | Make-up brand store |
|  | Bath & Body Works | Make-up brand store |
|  | Sephora (1) | Make-up brand store |
|  | Sephora (2, in JCPenney) | Make-up brand store |
|  | Aritaum, Amore Hannam | Make-up brand store |
|  | Walgreens | Drug store |
|  | Rite Aid | Drug store |
|  |  |  |
| Clinica de Salud del Valle de Salinas | Walmart | Big Box Store |
|  | Foods Co | Grocery Store |
|  | Food 4 Less | Grocery Store |
|  | Cardenas | Grocery Store |
|  | DC Fashion Mart | Local/Community Store |
|  | La princesa market | Local/Community Store |
|  | Indoor swap meet | Local/Community Store |
|  | Alisal Market | Local/Community Store |
|  | CVS | Drug store |
|  | Rite Aid | Drug store |
|  |  |  |
| Healthy Heritage Movement | PCH Beauty Supply | Beauty Supply Store |
|  | OK Beauty & Hair | Beauty Supply Store |
|  | Ulta | Beauty Supply Store |
|  | Sally Beauty Supply | Beauty Supply Store |
|  | All Star Beauty Supply | Beauty Supply Store |
|  | Target (1) | Big Box Store |
|  | Target (2) | Big Box Store |
|  | Walmart | Big Box Store |
|  | Food 4 Less | Grocery Store |
|  | CVS (1) | Drug store |
|  | CVS (2) | Drug store |

**GCxGC-TOFMS Analysis Sample Preparation.** Approximately 0.5 g of each sample was weighed, except for the one paper and one wipe product, for which approximately 5.5 g were utilized. Each sample was spiked with 50 μg of the isotopically labeled surrogate 1,4-dioxane-d8. The samples were Soxhlet extracted for 18 hours using 200 mL of methylene chloride (DCM). The sample extract was then concentrated using a high-purity nitrogen stream to a final effective volume of 10.0 mL. Each sample extract was further diluted 5-fold into DCM and spiked with deuterated internal standards to a level of 1 μg/mL each. The deuterated internal standards included 1,4-dichlorobenzene-D4, naphthalene-D8, acenaphthene-d10, phenanthrene-D10, chrysene-D12, and perylene-D12. After initial analysis, each data file was assessed for overloading and/or adequate response to determine if further dilution of the sample extract was warranted or if the sample extract required analysis at the undiluted level. Negative controls include solvent blanks prepared with extraction batch and DCM blanks assayed between each sample. Positive controls in addition to the surrogate and internal standards included reference standards assayed either using a calibration curve or as a retention-time standard for confirmation of identity (Tables S1 and S2). For three compounds (celestolide, versalide and lilial), a reference standard was not available. These compounds were measured in a second analysis using high resolution GCxGC-TOFMS, and the accurate mass of the fragmentation ions were consistent with the structure.

**GCxGC-TOFMS Instrument Details.** Two-dimensional gas chromatography time-of-flight mass spectrometric (GCxGC-TOFMS) analysis was performed using an Agilent 7890 gas chromatograph coupled to a LECO PEGASUS 4D-TOF (LECO, St. Joseph, MI). Injection volume was 1.0 µL. The inlet temperature was 275°C and the inlet mode was splitless with a 1 min purge. Separation was achieved using two columns. The primary column (1st Dimension) was an RXi-1MS (30 m × 0.25 mm × 0.25 µm; Restek, Bellefonte, PA) and the second column (2nd Dimension) was an RXi-17SilMS (1.7 m × 0.18 mm × 0.18 µm; Restek, Bellefonte, PA). The first column was held at 45°C for 3 minutes, ramped to 325°C at a rate of 7°C/min then held for 5 minutes. The second column and modulator were offset by 5°C and 20°C, respectively, relative to the primary oven. Helium carrier flow was set to constant flow at 1.0 mL/min. The transfer line temperature was 300°C. The modulation period was 7 seconds (1.75 s hot, 1.75 s cold with 2 cycles per modulation period). The mass spectrometer was operated using electron ionization (EI) at 70eV. The ion source temperature was set at 225°C. Spectra were collected from 45–650 m/z with a scan time of 100 spectra/sec.

High resolution GCxGC-TOFMS analysis was performed using an Agilent 7890A gas chromatograph coupled to a LECO HRT (LECO, St. Joseph, MI). Injection volume was 1.0 µL. The inlet temperature was 275°C and the inlet mode was splitless with a 1 min purge. Separation was achieved using two columns. The primary column (1^st^ dimension) was a RXi-1MS (30 m × 0.25 mm × 0.25 µm; Restek, Bellefonte, PA) and the second column (2^nd^ dimension) was a RXi-17SilMS, (1.3 m × 0.25 mm × 0.25 µm; Restek, Bellefonte, PA). The first column was held at 45°C for 3 minutes, ramped to 330 °C at a rate of 8°C/min, and held for 5.0 minutes. The second column and modulator were offset relative to the primary oven by 5.0 and 20°C, respectively. Helium carrier flow was set to constant flow at 1.2 mL/min. The transfer line temperature was set at 300°C. The modulation period was 4 seconds (1.0 s hot, 1.0 s cold with 2 cycles per modulation period). The mass spectrometer was operated using electron ionization (EI) at 70eV. The ion source temperature was set at 225°C. Spectra were collected from 45–650 m/z with a scan time of 100 spectra/sec.

**Table S2. Reference Standards with Calibration Curves (Target Analytes)**

| Name | CAS | Correlation Coefficient | RT | Lowest Standard Assayed (ppb) | Lowest Standard Observed (ppb) | Max Concentration Included in Curve (ppb) | Number of Calibration Points |
| --- | --- | --- | --- | --- | --- | --- | --- |
| 4-Nonylphenol | 104-40-5 | 0.999 | 1525 , 3.380 | 639 | 639 | 15985 | 5 |
| 4-Nonylphenol monoethoxylate | 104-35-8 | 0.994 | 1742 , 3.590 | 640 | 640 | 16000 | 5 |
| 4-tert-Octylphenol | 140-66-9 | 0.996 | 1280 , 3.440 | 638 | 638 | 15955 | 5 |
| 1,4-Dioxane | 123-91-1 | 0.996 | 195 , 2.410 | 200 | 200 | 10000 | 6 |
| Nitrobenzene | 98-95-3 | 0.999 | 664 , 4.520 | 200 | 500 | 10000 | 5 |
| Benzophenone-3 | 131-57-7 | 1.000 | 1651 , 5.230 | 200 | 5000 | 10000 | 2 |
| Methyl-benzylidene camphor (4-MBC) | 36861-47-9 | 0.999 | 1679 , 4.290 | 200 | 200 | 5000 | 6 |
| Octyl dimethyl PABA | 21245-02-3 | 0.999 | 1833 , 4.090 | 200 | 200 | 10000 | 6 |
| Octyl methoxycinnamate | 5466-77-3 | 0.999 | 1868 , 3.920 | 200 | 200 | 10000 | 6 |
| Bisphenol A | 80-05-7 | 0.982 | 1749 , 6.240 | 638 | 638 | 15940 | 6 |
| Triclosan | 3380-34-5 | 1.000 | 1707 , 4.810 | 2000 | 2000 | 10000 | 3 |
| Benzyl paraben | 94-18-8 | 0.980 | 1763 , 5.280 | 500 | 5000 | 10000 | 3 |
| Butylparaben | 94-26-8 | 0.999 | 1343 , 4.230 | 500 | 1000 | 10000 | 5 |
| Ethylparaben | 120-47-8 | 0.992 | 1189 , 4.460 | 500 | 2500 | 10000 | 4 |
| Heptyl paraben | 1085-12-7 | 1.000 | 1658 , 4.090 | 500 | 5000 | 10000 | 3 |
| Isobutylparaben | 4247-02-3 | 0.997 | 1392 , 4.260 | 500 | 1000 | 10000 | 5 |
| Isopropylparaben | 4191-73-5 | 1.000 | 1294 , 4.310 | 500 | 2500 | 10000 | 4 |
| Methylparaben | 99-76-3 | 0.990 | 1126 , 4.450 | 500 | 2500 | 10000 | 4 |
| Propylparaben | 94-13-3 | 0.989 | 1224 , 4.170 | 500 | 500 | 10000 | 6 |
| Benzyl butyl phthalate | 85-68-7 | 0.999 | 1875 , 5.260 | 200 | 200 | 10000 | 6 |
| Bis(2-butoxyethyl) phthalate | 117-83-9 | 0.999 | 1966 , 4.320 | 200 | 200 | 10000 | 6 |
| Bis(2-ethoxyethyl)phthalate | 605-54-9 | 0.992 | 1707 , 4.740 | 200 | 200 | 5000 | 5 |
| Bis(2-ethylhexyl) phthalate | 117-81-7 | 0.991 | 2029 , 3.440 | 200 | 200 | 10000 | 6 |
| Bis(2-methoxyethyl) phthalate | 117-82-8 | 0.997 | 1609 , 5.280 | 200 | 200 | 5000 | 5 |
| Bis(4-Methyl-2-pentyl)phthalate | 84-63-9 | 0.998 | 1693 , 3.430 | 200 | 200 | 5000 | 5 |
| Dibutyl phthalate | 84-74-2 | 0.994 | 1588 , 4.080 | 200 | 200 | 5000 | 5 |
| Dicyclohexyl phthalate | 84-61-7 | 0.995 | 1994 , 5.210 | 200 | 200 | 10000 | 6 |
| Diethyl Phthalate | 84-66-2 | 0.997 | 1252 , 4.560 | 200 | 200 | 10000 | 6 |
| Dihexyl phthalate | 84-75-3 | 0.999 | 1889 , 3.740 | 200 | 200 | 5000 | 5 |
| Diisobutyl phthalate | 84-69-5 | 0.984 | 1511 , 3.890 | 200 | 200 | 10000 | 6 |
| Dimethyl phthalate | 131-11-3 | 0.997 | 1105 , 5.020 | 200 | 200 | 10000 | 6 |
| Di-n-nonyl phthalate | 84-76-4 | 0.998 | 2260 , 3.610 | 200 | 200 | 10000 | 6 |
| Di-n-octyl phthalate | 117-84-0 | 0.998 | 2141 , 3.660 | 200 | 200 | 10000 | 6 |
| Dipentyl phthalate | 131-18-0 | 0.999 | 1742 , 3.910 | 200 | 200 | 10000 | 6 |

**Table S3.** **Chemicals confirmed using retention time (RT) standard or measured by high resolution MS**

| Name | CAS | RT | RT confirmed with reference standard | Measured by high resolution MS |
| --- | --- | --- | --- | --- |
| 7-Acetyl-6-ethyl-1,1,4,4-tetramethyltetralin (Versalide) | 88-29-9 | 1511 , 3.590 |  | X |
| Celestolide | 13171-00-1 | 1399, 3.17 |  | X |
| Lilial | 80-54-6 | 1196 , 3.600 |  | X |
| 2-Ethyl-1-hexanol | 104-76-7 | 615, 2.40 | X |  |
| Benzaldehyde | 100-52-7 | 496 , 4.080 | X |  |
| Benzoic acid | 60-12-8 | X790, 3.59 | X |  |
| Benzophenone | 119-61-9 | 1294, 4.94 | X |  |
| Benzyl chloride | 100-44-7 | 573, 3.75 | X |  |
| Bis(2-ethylhexyl) ester hexanedioic acid | 103-23-1 | 1945 , 2.730 | X |  |
| Butylated Hydroxytoluene | 128-37-0 | 1196 , 2.960 | X |  |
| Cyclohexanone | 108-94-1 | 391 , 3.780 | X |  |
| Estragole | 104-46-1 | 832, 3.38 | X |  |
| Ethylbenzene | 100-41-4 | 384 , 2.520 | X |  |
| Homosalate | 118-56-9 | 1546 , 3.430 | X |  |
| Methyl salicylate | 119-36-8 | 825, 3.68 | X |  |
| Musk ketone | 81-14-1 | 1602, 4.63 | X |  |
| Beta-myrcene | 123-35-3 | 566 , 2.170 | X |  |
| Pulegone | 89-82-7 | 874, 3.55 | X |  |
| Safrole | 94-59-7 | 937 , 3.740 | X |  |
| Tonalid | 21145-77-7 | 1525 , 3.430 | X |  |
